# New Year’s Address

**Published:** 1848-03

**Authors:** 


*New Year's Address.*—Being absent from our editorial post, New Year's day was allowed to pass without the usual greeting to our readers and patrons. It is too late to redeem the omission, of which we were reminded by the following short, but pithy address by our much esteemed, and able contemporary, the New Orleans Medical and Surgical Journal:—" The dawn of a new year, we deem a fit occasion to admonish our friends that if they *really wish* a Medical Journal from the *Emporium* of the South, they must lend us more efficient aid than they have hitherto done. They must *write more*, *pay better*, and afford us *a larger subscription list*," Will our readers make a personal application of the address, substituting for the *Emporium of the South* the *Emporium of the West!*

